# Olive Oil Nutraceuticals in the Prevention and Management of Diabetes: From Molecules to Lifestyle

**DOI:** 10.3390/ijms19072024

**Published:** 2018-07-12

**Authors:** Ahmad Alkhatib, Catherine Tsang, Jaakko Tuomilehto

**Affiliations:** 1 Dasman Diabetes Institute, Kuwait P.O. Box 1180, Dasman 15462, Kuwait; jaakko.tuomilehto@dasmaninstitute.org; 2 Faculty of Health and Social Care, Edge Hill University, St. Helens Road, Ormskirk, Lancashire L39 4QP, UK; Tsangc@edgehill.ac.uk

**Keywords:** olive nutraceuticals, functional foods, exercise, nutrition, type-2 diabetes

## Abstract

Lifestyle is the primary prevention of diabetes, especially type-2 diabetes (T2D). Nutritional intake of olive oil (OO), the key Mediterranean diet component has been associated with the prevention and management of many chronic diseases including T2D. Several OO bioactive compounds such as monounsaturated fatty acids, and key biophenols including hydroxytyrosol and oleuropein, have been associated with preventing inflammation and cytokine-induced oxidative damage, glucose lowering, reducing carbohydrate absorption, and increasing insulin sensitivity and related gene expression. However, research into the interaction of OO nutraceuticals with lifestyle components, especially physical activity, is lacking. Promising postprandial effects have been reported when OO or other similar monounsaturated fatty acids were the main dietary fat compared with other diets. Animal studies have shown a potential anabolic effect of oleuropein. Such effects could be further potentiated via exercise, especially strength training, which is an essential exercise prescription for individuals with T2D. There is also an evidence from in vitro, animal, and limited human studies for a dual preventative role of OO biophenols in diabetes and cancer, especially that they share similar risk factors. Putative antioxidative and anti-inflammatory mechanisms and associated gene expressions resulting from OO biophenols have produced paradoxical results, making suggested inferences from dual prevention T2D and cancer outcomes difficult. Well-designed human interventions and clinical trials are needed to decipher such a potential dual anticancer and antidiabetic effects of OO nutraceuticals. Exercise combined with OO consumption, individually or as part of a healthy diet is likely to induce reciprocal action for T2D prevention outcomes.

## 1. Introduction

Diabetes is a major health problem and one of the leading causes of morbidity and mortality worldwide [1]. The current estimated prevalence has already reached over 400 million people [2]. Preventing type 2 diabetes (T2D) is possible mainly through lifestyle adjustments. Large prospective studies have all shown remarkable reductions in T2D incidence through combinations of dietary and physical activity modifications [3,4]. Reduced T2D incidence rates have also been found more recently in the PREDIMED follow up study, which also demonstrated key benefits of the Mediterranean diet (MD) such as adherence in reducing cardiovascular disease and mortality rates [5,6]. Such interest has made it important to review the T2D preventative role of functional foods and bioactive components present within MD including vegetables and fruit, olive oil, fish, and tree nuts [7]. Given that olive oil (OO), especially in its extra-virgin form, is the distinct macronutrient lipid and key culinary ingredient characterizing MD, it would be important to review the T2D preventative bioactive ingredients of OO from molecular to whole body level.

At a molecular level, several bioactive ingredients within OO have been repeatedly linked with anti-oxidant and anti-inflammatory preventative functions, particularly those from monounsaturated fatty acids (MUFA), and key biophenols such as oleuropein and hydroxytyrosol (HT) [8]. The health benefits of OO in T2D prevention and management continues to be of a growing research interest and a simple search on PubMed using OO and diabetes as keywords revealed 417 entries, and this was increased to 1667 entries with the search of OO and health. Readers can also refer to recent systematic reviews for virgin OO effects in T2D prevention [9]. However, there is a lack of research on how OO and its phenolic components function as part of lifestyle prevention of T2D, especially when combined with enhanced physical activity. Augmenting the benefits of healthy nutritional food components, or functional food, with adding lifestyle approaches such as exercise, can extend a comprehensive model for T2D prevention and management previously presented [7]. This review aims to investigate key molecular components of OO ingredients and how they interact with lifestyle approaches to prevent disease, especially T2D. It will also discuss recent findings of novel molecular functions of OO, and how they can be augmented in the lifestyle prevention of T2D and associated diseases.

## 2. Bioactive Compounds and Key Functions of Olive Oil: Relevance for Diabetes

Over 30 hydrophillic biophenolic compounds have been identified in OO derived from the olive tree fruit (*Olea europaea* L., Oleaceae family), most of which are responsible for the organoleptic properties, bitter and pungent flavours and aromas, and oxidative stability of the oil [10,11,12]. Biophenols are a diverse and heterogenous group of compounds characterized by an aromatic benzene ring attached to one or more hydroxyl groups in their structure. They are synthesized as secondary plant metabolites via the shikimate, polyketide, and acetate biosynthetic pathways, producing C6–C3, and C6–C3–C6 derivatives, and aromatic terpenoids, respectively [13]. Several enzymatic transformations including condensation, cyclisation, glycosylation, hydroxylation, acylation, methylation, and prenylation contribute to the structural diversity of phenylalanine-derived metabolites [14] and tyrosol (4-(2-Hydroxyethyl) phenol), one of the major phenylethanoids derived from OO, which has the ability to form esters with fatty acids [15]. Levels of biophenols in OO are highly variable and are influenced by several factors including different varietal cultivars, degree of fruit ripening, stage of maturation, storage conditions, and processing methods [16,17]. Nonetheless, studies have shown that extra virgin OO (EVOO) contains greater levels of biophenols (ca. 50–800 mg/kg) compared with those of refined OO (ca. 62–198 mg/kg), which undergo further and more extensive processing [18]. HT (3,4-DHPEA) and tyrosol (p-HPEA) comprise over 90% of the total phenolic content of OO, in addition to their secoiridoid derivatives—dialdehydic forms of elenolic acid (EA) linked to HT (oleacein: 3,4-DHPEA-EDA) and tyrosol (oleocanthal: p-HPEA-EDA), aglycones of oleuropein (3,4-DHPEA-EA) and ligstroside (p-HPEA-EA). Hydrophilic esters of EA; tyrosol, HT, oleocanthal, and oleuropein, and their associated compounds; 10-hydroxyoleuropein, ligstroside, and 10-hydroxyligstroside are the most prevalent [19]. Lignans; (+)-1-acetoxypinoresinol and (+)-1-hydroxypinoresinol, and their respective glucosides have been detected in the bark of the olive tree, and in OO, and levels have been reported to be in the region of ca. 100 mg/kg [20]. Phenolic acids; sinapic, vanillic, caffeic, ferulic, p-hydroxybenzoic, p-coumaric acid, protocatechuic acid, and hydroxy-isocromans; 1-phenyl-6,7-dihydroxy-isochroman and 1-(3′-methoxy-4′-hydroxy)-6,7-dihydroxy-isochroman, synthesized from reactions with HT, benzaldehyde, and vanillin, have also been detected in OO, however levels rarely exceed ca. 1 mg/kg [21]. Similarly, flavonoids, luteolin and apigenin are present in levels much lower in comparison to other phenolics derived from OO [22]. HT, tyrosol, and oleuropein are of scientific interest due to their significant effects on several molecular, genetic, and biological mechanisms, which could contribute to the prevention of chronic diseases such as T2D.

Several important biological properties have been ascribed to biophenols derived from OO, including antioxidant; free-radical scavenging and cardio-protective effects, and their ability to modulate pro-inflammatory cytokines and markers of inflammation, which could mitigate modifiable risk factors associated with T2D [23,24,25]. The cardioprotective role of HT and their derivatives, particularly oleuropein, in their ability to improve high-density lipoproteins (HDL) [26], reduce low-density lipoprotein (LDL), inhibit platelet aggregation, and improve endothelial function [27] are well recognized. Health claims exist in the EU for the role of OO derived biophenols in their protection against the oxidation of blood lipids, and maintenance of normal blood LDL-cholesterol levels [28], and current recommendations suggest a daily intake of ca. 20 g of EVOO (of which 5 mg is derived from HT and its derivatives) to protect from CVD [29]. Such recommendations do not yet exist for T2D. Evidence from in vitro, in vivo, and clinical studies indicate significant anti-inflammatory effects of HT in their ability to reduce the expression of adhesion and signaling molecules and inflammatory markers [30,31]. These effects are well documented in those at risk for CVD; however, few studies have been tested in people with or at risk of T2D. Nonetheless, OO derived biophenols may reduce postprandial inflammation by decreasing the activation of nuclear-factor kappa B (NF-κB) and lipopolysaccharide (LPS) absorption. Camargo et al. [32] administered a virgin OO enriched meal with different concentrations of phenolics (High: 398 ppm, Intermediate: 149 ppm and Low: 70 ppm) to subjects with metabolic syndrome (MetS) including T2D. Inhibition of NF-κB and decreased expression of interleukin-IL-1β and IL-6 was observed following the meal enriched with the highest concentration (398 ppm) of OO. Reduced fasting plasma glucose concentrations, glycated haemoglobin A1c (HbA1c), body weight, and inflammatory adipokines have also been demonstrated in a small-scale study with overweight T2D patients following intake of EVOO (equivalent to 577 mg/kg, mainly as HT) [33]. Phenolics could exert potential anti-diabetic effects due to their potent free-radical scavenging and antioxidative properties. Animal models and in vitro evidence demonstrate their interaction with intracellular signaling pathways, such as nuclear transcription factor (erythroid-derived 2)-like 2 (Nrf2), which is involved in the regulation of the expression of antioxidant proteins that protect against oxidative damage. In vitro studies indicate the potential role of HT and oleuropein in their ability to protect cells against oxidative stress by activating the Nrf2/ARE pathway in a dose-dependent manner, with HT exhibiting potent radical scavenging capacity [34] and ability to upregulate protective enzymes including thioredoxin reductase [35]. Evidence from a recent meta-analysis on OO consumption in T2D patients reported a lower production of advanced glycosylated end-products (AGE’s) [9]. HT supplementation (10 mg/kg/day for 5 weeks) enhanced glucose tolerance and insulin sensitivity leading to a decrease of homeostatic model assessment-insulin resistance in rat models [36]. Further potential anti-diabetic mechanisms have been demonstrated in experimental in vitro studies for flavonoids and phenolic acids e.g., chlorogenic, ferulic, caffeic, and tannic acids, in their ability to inhibit α-amylase, α-glucosidase enzymes, and the sodium dependent SGLT1-mediated glucose transport, thus potentially influencing glucose metabolism by inhibiting carbohydrate digestion and absorption [37,38]. HT has a high degree of bioavailability as evidenced by their high rates of absorption following ingestion of EVOO (40–95%) in humans; oleuropein-glycoside, oleuropein, and ligstroside–aglycones are converted to HT or tyrosol and excreted in urine, and HT and tyrosol themselves are sometimes conjugated to glucuronic acid and excreted in urine as glucuronides [39]. It is also thought that ingestion in this formulation (i.e., oil) could further mitigate their breakdown in the gastrointestinal tract. The mechanisms of absorbing and exerting key OO bioactive compounds may explain its fate and preventative effects in T2D and other cardiometabolic diseases. It is likely that biophenols may influence glucose metabolism via several mechanisms; inhibition of carbohydrate digestion and glucose absorption in the intestine, activation of insulin receptors and glucose uptake in the tissues, antioxidative properties, and immunomodulatory effects.

Several T2D protective mechanisms of OO and similar olive leaves biophenols have been reported from cell culture, animal and human studies, and summarized in detail elsewhere [40]. Those include oleuropein effects on reducing amyloid aggregation and preventing inflammation and cytokine-induced oxidative damage of pancreatic β-cells and enhancing β-cells capacity; olive leaves extracts effects include lowering glucose and cholesterol levels; modifying gene expression implicated in lipogenesis, thermogenesis and insulin resistance; reducing digestion and intestinal absorption of dietary carbohydrates in the mucosal and in serosal sides of the intestine; reducing HbA1c and fasting plasma insulin; acutely enhancing insulin sensitivity and related gene expression by OO ingestion [41]; oleacein preventing inflammatory response and cytokine-mediated oxidative cell damage with downregulation of a number of genes involved in adipocyte differentiation. It is however unclear at this juncture, the precise role of OO biophenols, and further investigations, especially in humans, are necessary to fully elucidate their mechanisms in T2D.

## 3. Does Olive Oil Prevent T2D Independently or as Part of a Healthy Diet?

Attributing health benefits to OO cannot be investigated in isolation of other healthy dietary and lifestyle components, especially since OO has been the defined food component characterizing MD, which contains other healthy foods such as seafood, fruits and vegetables, and nuts [42]. However, longitudinal prospective studies which OO to households who consumed MD reported better cardioprotective outcomes compared with MD supplemented with nuts, despite both diets showed better risk-reduction outcomes than low-fat control diet [5]. In the 10-year follow-up of this study, T2D incidence was lower with OO supplemented MD compared with MD supplemented with nuts or low-fat diet control (80, 92, and 101), and this corresponded to lower hazard ratios (0.60 vs. 0.82) in the MD supplemented with OO compared with the MD with nuts [6]. Another longitudinal study also reported a lower 10-year incidence of T2D and CVD events in prediabetic individuals who had a higher adherence to MD components [43]. However, OO was part of 11 other components in the 55-score Greek MD scales used in the latter compared with OO being part of 9 other components in the Spanish 14-item MD scale used in the PREDIMED follow-up study [6,44]. Other MD interventions have also shown relevant improvement in T2D biomarkers and enhanced microvascular and cardiorespiratory outcomes when MD was combined with an 8-week exercise intervention and a one-year follow up, with OO being the key ingredient implemented as part of the MD 9 components in older adults and postmenopausal women cohorts [45,46,47]. MD-induced enhancement in endothelial function and markers of vascular inflammation has been associated with improved glucose tolerance in individuals with MetS [48].

In a sub-group of the PREDIMED study follow up (after 1 year), the cardioprotective anti-inflammatory benefits were attributed to OO only based on lower plasma tumor necrosis factor receptor (TNFR60) concentration found in individuals allocated in the highest tertile of OO and vegetables consumption compared with those in lowest tertile [49]. This was combined by an overall MD-components induced reduction in plasma IL-6, TNFR60, and TNFR80, compared with an increase in those who followed a low-fat diet. Thus, there is convincing evidence that consuming OO as part of a healthy MD diet is protective of T2D in high-risk individuals with prediabetes and those with high CVD risk.

The current evidence from cohort studies on OO in T2D prevention stems from meta-analyses, which have shown that OO is the key MUFA (55–80% oleic acid), and that MUFA from vegetable sources are responsible for alleviating T2D metabolic risk factors and reducing all-cause mortality, stroke, and CVD events [9,25]. Nevertheless, evidence from randomized controlled trials has often focused on testing the main OO biophenol compounds (oleuropein, tyrosol, HT, flavonoids, and lignans) and other MUFA compounds. These compounds have been reviewed recently for their effectiveness in the prevention of T2D, especially showing increased HDL, enhanced endothelial vascular activity, reduced reliance on carbohydrate substrates, and reduced glucose release from the liver, as well as increased glucose uptake in peripheral tissues, which can reduce HbA1c [9]. When compared with other healthy oils with similar constituents such as a-linoleic acid in rapeseed oil (common in the Nordic diet), OO biophenols, especially oleuropein, have been suggested to be superior in their anti-oxidation and effects on blood lipids [50]. For example, the EPIC-Interact study has shown that phospholipid α-linoleic acid (a compound found only in oleic acid of OO) is inversely associated with T2D [51]. Further anticancer and cardioprotective properties within α-linolenic acid of OO have been suggested to be superior than α-linoleic acid found in rapeseed oil, especially when such oils are consumed as part of a healthy diet such as MD [9,50].

## 4. New Scope for Olive Oil, Physical Activity, and Lifestyle Approaches in T2D Prevention

The synergistic effects between healthy food components including OO and other lifestyle factors, especially physical activity, is integral to the prevention and management of T2D. Better T2D outcomes can be achieved, whether through combining nutritional ingestions with exercise, or with other lifestyle approaches to augment the mechanistic preventative effects of functional foods (i.e., molecular, metabolic, vascular, and behavioral), and has been shown to be effective as part of a model we recently developed for the prevention and management of T2D [52].

However, only a limited number of well-designed studies have tested the effectiveness of OO synergy with exercise interventions on relevant T2D outcomes. For example, combining 1-h of moderate endurance exercise with consuming an OO breakfast meal (saturated fat 15% and unsaturated fat 85%) produced a 26% lower postprandial triglyceride (TG) than a butter-no exercise meal (saturated fat 71% and unsaturated fat 29%) [53]. Such combined effects suggest positive mechanisms for OO on lipid abnormalities associated with T2D or “diabetic dyslipidemia” such as the high concentration of TG and small dense LDL and a low concentration of HDL cholesterol [54]. Another study using animal models has shown that diets with OO induced better counteracting benefits to exercise-induced oxidative-stress (26% vs. 17% increase in the area under the curve) compared with a butter-based diet trial [55]. This suggests that the benefits of OO may be significantly augmented when combined with exercise, due to reciprocal actions on T2D outcomes and exercise-induced oxidative-stress. Other studies encompassing EVOO as part of MD have also shown effectiveness in combining MD with supervised moderate exercise training in enhancing microcirculatory vascular activity in high-risk individuals [45,46]. A 6-month multicomponent lifestyle school-based program consisting of four different lifestyle approaches (physical activity, nutrition education, combined with substituting normally taken oil with EVOO) found that glycemic and diastolic blood pressure were reduced in the intervention group who adopted EVOO [56]. OO consumption as part of a health dietary plan is likely to produce better T2D prevention outcomes when combined with exercise, and requires further investigations.

Enhancing the anabolic effects of strength training through novel effects of OO compounds is another interesting area in T2D prevention, especially given the importance of strength training for patients with T2D patients or those at high-risk [2]. Recent evidence from animal studies indicated novel anabolic enhancing effects of OO biophenols on androgen function. For example, oleuropein supplementation increased testicular testosterone concentrations, reduced plasma corticosterone, and enhanced plasma LH in rat models [57]. These anabolic effects were observed following the addition of 0.1 g per 100 g oleuropein to a high protein (40%) diet (40, 25, and 10 g per 100 g casein) levels for 28 days high-protein diet [58].

Oleuropein has also been found to confer a higher resistance to oxidation of EVOO when compared with other healthy oils such as rapeseed oil [50,51]. Whether and how oleuropein potentiates anabolic effects can be enhanced via exercise training is yet to be investigated in T2D prevention.

For example, testosterone deficiency promotes insulin resistance and increases the risk of T2D [59]. Testosterone plays a critical role in the regulation of body composition in males and exhibits potential anti-obesity effects mediated by the androgen receptor (AR) [60]. Emerging research from knockout mice indicates a protective mechanism of AR signaling in adipocytes, critical in the regulation of insulin action and glucose homeostasis, independent of adiposity [61]. This new insight into the importance of AR activity may potentially lead to the development of a new multicomponent lifestyle strategy targeted at insulin resistance associated with testosterone deficiency, for which OO could play an important therapeutic role.

In addition to their potential anabolic effects, OO biophenols may play a role in augmenting strength training outcomes as part of T2D prevention via their putative added modulation of the anti-inflammatory, anti-oxidation, and pro-hypertrophy mechanism, especially when OO was ingested as part of a dietary plan. A recent rodent study reported an increase in muscle hypertrophy, articular cartilage recovery, and reduced IL-6 in rats with early osteoarthritis when exercise (daily treadmill running 5 days a week for 10 min) was combined with ingesting a standardized diet enriched with EVOO for 12 weeks [62]. The combined anti-inflammatory and pro-hypertrophy mechanism induced by conjugated OO jointly with exercise could be effective in preventing and treating T2D and associated complications. For example, reducing inflammatory cytokines could counteract muscle catabolism via actions on monocyte adhesion proteins such as monocyte chemoattractant protein-1 molecule (MCP1) [63].

In the context of lifestyle T2D prevention, effects of OO are not exclusive to exercise and diet. Disease-related detriments to other lifestyle behaviors such as sleep disturbance, fatigue, depression, and stiffness have also been shown to improve with OO combined with exercise intervention in women with fibromyalgia [64]. Such positive synergetic effects of OO and exercise could be explained by their effect on oxidative stress, especially on reducing inflammatory cytokines IL-6 and TNF-α [65], which are key biomarkers in T2D prevention and management. Further research is needed to test such synergetic effects in high-risk and T2D individuals. Research in this area is especially important given the promising evidence for augmented T2D preventative effects, when physical activity is combined with selected functional foods and nutraceutical of MD and naturally available health promoting herbs, as summarized elsewhere [7].

Another interesting lifestyle approach is to investigate whether biophenols, including those in OO can augment physiological exercise performance, especially cardiorespiratory exercise capacity. Enhanced cardiorespiratory fitness is known to associate with disease prevention especially cardio-metabolic disorders [66]. However, a recent review did not provide convincing evidence about the role of biophenols and exercise performance [67]. Quercetin supplementation for 7 days was reported to enhance aerobic capacity and improve exercise time, but the meta-analysis relied on a small number of studies, which used non-OO biophenols and may not apply to this review. Nonetheless, enhancing exercise-dependent outcomes, especially cardio metabolic, through OO supplementation is potentially increasing its effectiveness for T2D prevention when adopted as part of a lifestyle approach.

## 5. Diabetes, Cancer Mechanisms and Olive Oil Interrelationship

The link between hyperinsulinemia, T2D, and cancer is a research area of a growing interest, since there is evidence that people with diabetes are at a significantly higher risk of developing many forms of cancer given their similarities in risk factors and pathophysiology [68]. Some evidence indicates a higher risk of more aggressive and metastatic forms of cancer, with poor prognosis in diabetics [68,69]. Plausible biological mechanisms have been described to account for this link including the effects of hyperglycemia, hyperinsulinemia, and inflammation on cancer etiology and progression [70]. Insulin is a growth factor, which stimulates cell mitosis and migration, and inhibits apoptosis, effects that could potentially become exacerbated under conditions of insulin resistance and impairment of insulin-regulated metabolic pathways, as seen in T2D.

Therefore, the potential of OO as a protective agent in both diabetes and cancer makes it interesting to decipher their underlying mechanisms, and to pave the way to develop effective treatment approaches. Evidence from epidemiological studies indicate a potential role of OO in the prevention of certain cancers, especially those affecting the breast and colon [71,72,73,74]. Potential anti-cancer effects of OO biophenols have been shown in experimental studies, whereby oleuropein inhibited cancer cell growth and induced apoptosis in human breast cancer cell lines, T-47D, and MCF-7 via the p53-dependent pathway and via regulation of *Bax* and *Bcl2* genes [75,76]. Similarly, HT reduces hydrogen peroxide induced DNA damage in human peripheral blood mononuclear cells and promyelocytic leukemia cells (HL60) [77]. The prevention of ROS-induced DNA damage is a potential mechanism of defense against the multistage process of carcinogenesis, and DNA mutations arising from damage caused to DNA is a common feature in carcinogenesis. Pathophysiological manifestations of diabetes, i.e., increased plasma glucose, insulin, AGEs and free fatty acids, enhanced reactive oxygen species (ROS), and oxidative stress, and increased DNA damage, have been reported to be considerably higher in people with poor glycemic control, and diabetes [78,79]. Potential anticancer and antidiabetic effects of OO biophenols are likely mediated, in part, by their potent antioxidant and free radical scavenging properties, and human intervention studies albeit limited, have shown decreased levels of urinary 8-oxo-7,8-dihydro-2′deoxyguanosine (8-OHdG), a known biomarker of DNA damage, after short-term consumption of OO [80].

Secoiridoid from OO especially oleocanthal, have been shown to inhibit the proliferation, migration, and invasion of various human breast, prostate cancer, and multiple myeloma cells [81]. Oleocanthal is the OO compound responsible for the pungent sensation at the back of the throat, is thought to exert similar non-steroidal anti-inflammatory activity to that within ibuprofen, especially in inhibiting harmful cyclooxygenase (COX) 1 and COX2 enzymes [82]. Inhibition of COX2 and matrix metalloproteinases through OO biophenols oleuropein and HT have shown reduced angiogenesis in cultured endothelial cells [83]. Differences in the gene expression profile of breast tissue has also been reported in an animal model of breast cancer susceptibility following ingestion of OO compared with corn oil. Expression of metabolism genes related to mitochondrial uncoupling proteins, were found only after OO ingestion, suggesting a reduction in the balance of intake and expenditure, alongside a down-regulation of the expression of S100 genes [84] that have been associated with the progression of breast tumorigenesis. The inflammatory transduction of S100 protein signaling is mediated by receptor for advanced glycation end-products (RAGE), in a variety of cell types [85]. RAGE is a multi-ligand cell-surface receptor that propagates cellular dysfunction in several inflammatory disorders, in tumors and in diabetes [86]. It is also a marker for oxidative stress through its interaction with AGEs, where its accelerated formation due to increased concentration of circulating glucose is a feature of T2D [87]. The associated metabolic abnormalities between diabetes and cancer is significant and of clinical importance, and therefore, mandatory counselling and/or screening for changes linked with cancer could be one strategy to accompany lifestyle approaches in patients presenting with obesity, pre-diabetes, and diabetes.

Evidence from in vitro, animal, and limited human studies suggest potential benefit of OO biophenols via putative anti-oxidative and anti-inflammatory mechanisms involving NF-κB inhibition with COX-2, IL-6, IL-8, and IL-1β (down-stream products of NF-κB) expressed at lower levels. These may account for the lower prevalence of cancer in people consuming a MD. However, there is a long way still before such mechanisms are deciphered for each disease. For example, improving insulin sensitivity by inducing the inhibition of NFkB expression is also thought to mediate muscle wasting seen with disuse, denervation, and some systemic diseases (i.e., cancer, sepsis) [88]. High phenolic content OO has been shown to inhibit NF-κB and decrease IL-1β and IL-6 postprandially in individuals with MetS [31]. Clinical evidence showing dual antidiabetic and anticancer effects is limited and somewhat inconclusive, however intervention studies have reported some benefit of OO mostly based on changes in biomarkers associated with immunomodulatory and anti-oxidative capacity in healthy, diabetic, and cancer patients [32,49,80,89,90] (Table 1). However, these findings remain inconsistent and this could be due in part to a lack of robust and well-designed clinical trials.

## 6. Conclusions

**Figure 1:**
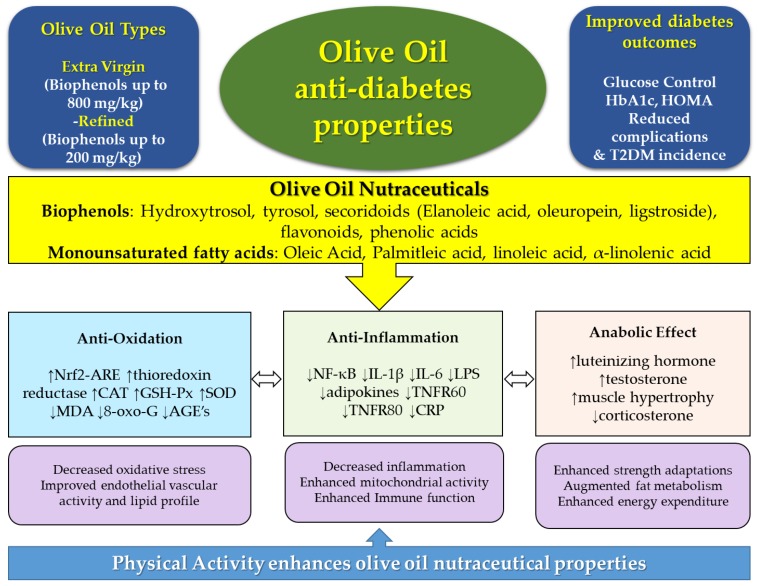
Olive oil, types, and phenolic compounds, and associated diabetes protective molecular mechanisms, which can potentially be augmented with physical activity. AGE’s: Advanced glycated end-products; CAT: catalase; CRP: c-reactive protein; Hb1Ac: glycated haemoglobin; GSH-Px: glutathione peroxidase; IL: interleukin; LPS: lipopolysaccharide; MDA: malonaldehyde; SOD: superoxide dismutase; TNFR: tumor necrosis factor receptor. Arrows within caption indicate decrease (↓) or increase (↑).

Lifestyle prevention of T2D necessitates investigating nutritional dietary bioactive compounds. OO intake as part of the diet has been associated with the prevention and management of T2D. OO contains an abundance of biophenols; oleuropein, HT and their derivatives, and several antidiabetic mechanisms have been ascribed to their potential immunomodulatory, antiproliferative, antioxidative, and anabolic effects. There is a promising evidence that such effects can be further augmented with combining physical activity lifestyle components with OO consumption. OO mechanisms have mainly emanated from in vitro studies and animal models, with limited clinical studies. Nonetheless, their potential effects on T2D and associated comorbidities is encouraging, such as the potential dual diabetes and cancer protective role found in OO nutraceuticals (Figure 1). Robust human intervention and clinical trials are necessary to fully elucidate the role of OO in T2D and their associated comorbidities, especially when combined with exercise.

## Figures and Tables

**Table 1:** Potential antidiabetic and anticancer dual effects of olive oil in human studies.

Reference	Patients	Dose and Formulation	Outcomes
Carmargo et al. [32]	*n* 49 with MetS, age range: 36–71 years old (19 men, 30 women); mean BMI: 38.59 ± 0.58 kg/m^2^	40 mL VOO intake over 24 h, provided as a breakfast of high (398 ppm), intermediate (149 ppm) or low (70 ppm) TP	High dose: Decrease NF-κB, IL-6, TLR4 protein, IL-1β expression Low dose: Increase NF-kB p65 subunit, IL-6; TLR4 protein, TNF-α.
Urpi-Sarda et al. [49]	*n* 106 sub-cohort at high risk of CVD, from the PREDIMED trial	VOO (1 L/week) compared with a control low-fat diet at 3 months and 1 year old follow-up	At 3 months: Reduced IL-6 and CRP with VOO At 1 y: Reduced TNFR 60, IL-6, TNFR80 Increase: IL-6, TNFR60, TNFR80 with low-fat diet
Weinbrenner et al. [80]	*n* 12 Healthy men, age range: 20–22 y, mean BMI: 22.9 ± 1.7 kg/m^2^	25 mL/day VOO: Subjects received 1 of the 3 treatments (25 mL/d) over 4 days with a washout period of 10 d between treatments. low, moderate and high TP content (10–486 mg/kg TP)	Decrease: 8-oxo-dG in mitochondrial DNA and urine, MDA in urine Increase: GSH-Px No effect: GR
De Bock et al. 2013 [89]	*n* 46 Overweight patients, mean BMI: 28.0 ± 2.0 kg/m^2^	OLE provided as capsules containing 51.1 mg oleuropein and 9.7 mg HT	28% Increase Beta cell function Increase: IL-6 No effect: IL-8, TNF-α, high-sensitive CRP
Oliveras-López et al. [90]	*n* 45 healthy men and women (age: 21–45 years old), mean BMI: 21.4 ± 0.5 kg/m^2^	50 mL EVOO for 30 days, two doses ingested at breakfast (30 mL) and lunch (20 mL)	Increase: Plasma AOX capacity, AOX enzymes—CAT, GPX; improved gene expression SOD

AOX: antioxidant; VOO: virgin olive oil; EVOO: extra virgin olive oil; OLE: olive oil leaf extract; 8-oxo-dG: 8-oxo-7,8-dihydro-2′deoxyguanosine; GR: glutathione reductase; HT: hydroxytyrosol; GSH-Px: glutathione peroxidase; IL-6: interleukin-6; IL-1B: interleukin-1beta; CRP: C-reactive protein; MDA: malonaldehyde; NF-κB: nuclear factor kappa B; ROS: reactive oxygen species; SOD: superoxide dismutase; TLR4: toll-like receptor 4; TNF-α: tumor necrosis factor-alpha; TP: total phenolics.

## Abbreviations

AGEs	Advanced glycosylated end products
AR	Androgen receptor
ARE	Antioxidant response element
Bax, Bc12	Apoptosis regulator genes
COX	Cyclooxygenase
CRP	C-reactive protein
CVD	Cardiovascular disease
EA	Elenolic acid
GSH-Px	Glutathione peroxidase
HbA1c	Haemoglobin A1c
HDL	High-density lipoprotein
HT	Hydroxytyrosol
HL60	Leukemia cell line
IL	Interleukin
LDL	Low-density lipoprotein
LPS	Lipopolysaccharide
T2D	Type 2 diabetes
OO	Olive oil
8-OHdG	8-oxo-7,8-dihydro-2′-deoxyguanosine
MCF-7	Breast cancer cell line
MCP1	Monocyte chemoattractant protein-1
MD	Mediterranean diet
MDA	Malonaldehyde
MetS	Metabolic syndrome
MUFA	Monounsaturated fatty acids
NF-κB	Nuclear-factor kappa B
Nrf2	Nuclear transcription factor (erythroid-derived-2)-like 2
PMBC	Peripheral blood mononuclear cells
P53	Tumor protein antigen
RAGE	Receptor for advanced glycation end products
ROS	Reactive oxygen species
SGLT-1	Sodium dependent mediated glucose transporter
SOD	Superoxide dismutase
TNFα	Tumor necrosis factor alpha
TNFR60	Tumor necrosis factor receptor
T-47D	Breast cancer cell line
